# The Relationship between Protein–Protein Interactions and Liquid–Liquid Phase Separation for Monoclonal Antibodies

**DOI:** 10.1021/acs.molpharmaceut.3c00090

**Published:** 2023-04-11

**Authors:** Nicole Sibanda, Ramesh Kumar Shanmugam, Robin Curtis

**Affiliations:** † Manchester Institute of Biotechnology, Department of Chemical Engineering, Faculty of Science and Engineering, The University of Manchester, Manchester M1 7DN, U.K.; ‡ Biopharmaceutical Development, Dosage Form Design and Development, AstraZeneca, Aaron Klug Building, Granta Park, Cambridge CB21 6BH, U.K.

**Keywords:** osmotic second virial coefficients, diffusion coefficients, formulations

## Abstract

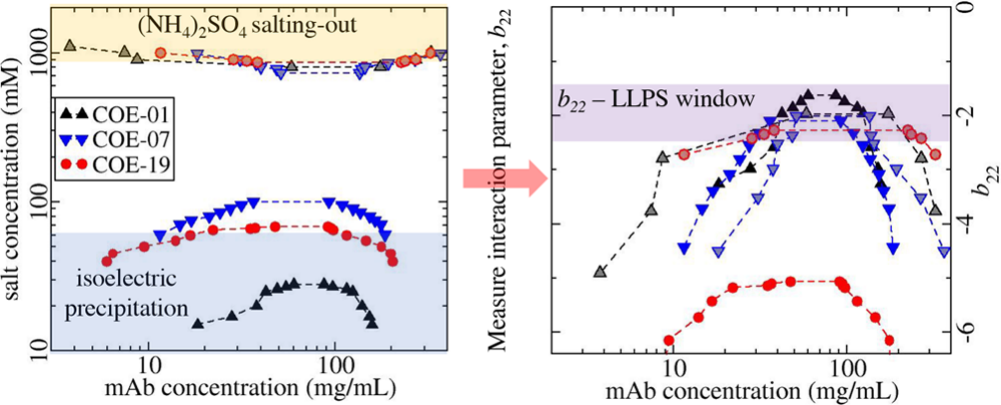

Being able to predict and control concentrated solution properties for solutions of monoclonal antibodies (mAbs) is critical for developing therapeutic formulations. At higher protein concentrations, undesirable solution properties include high viscosities, opalescence, particle formation, and precipitation. The overall aim of this work is to understand the relationship between commonly measured dilute solution parameters, the reduced osmotic second virial coefficient *b*_22_ and the diffusion interaction parameter *k*_D_ and liquid–liquid phase separation, which occurs at higher protein concentrations. For globular proteins such as lysozyme or γB-crystallin, the location of the liquid–liquid coexistence curve is controlled by the net protein–protein interaction, which is related to *b*_22_. Because many mAbs undergo reversible self-association due to forming highly directional interactions, it is not known if *b*_22_ can be used as a reliable predictor for LLPS since increasing the anisotropy in the interaction potential causes phase separation to occur at much stonger net protein–protein attractions or lower values of *b*_22_. Here, we map the coexistence curves for three mAbs, referred to as COE-01, COE-07, and COE-19, in terms of *b*_22_ and *k*_D_ values. The measurements are carried out at a low salt condition near the pI, where protein–protein interactions are expected to be anisotropic due to the presence of electrostatic attractions, and under salting-out conditions at high ammonium sulfate concentrations, which is expected to reduce the anisotropy by screening electrostatic interactions. We also show that deviations from a linear correlation between *b*_22_ and *k*_D_ can be used as an indicator of reversible self-association. Each of the mAbs under salting-out conditions follows the correlation supporting the hypothesis that protein–protein interactions are nonspecific, while deviations from the correlation occur for COE-01 and COE-19 under low salt conditions indicating the mAbs undergo reversible self-association. For five out of the six conditions, the onset of phase separation, as reflected by the reduced virial coefficient at the critical point *b*_22_^c^ occurs in a small window −1.6 > *b*_22_^c^ > −2.3, which is similar to what has been observed for lysozyme and for bovine γB-crystallin. Under low salt conditions, *b*_22_^c^ ≈ −5.1 for COE-19, which we previously showed to self-associate into small oligomers. Our findings suggest that under conditions where mAb interactions are weakly anisotropic, such as occur at high salt conditions, phase separation will begin to occur in a small window of *b*_22_. Deviations from the window can occur when mAbs undergo reversible self-association, although this is not always the case and likely depends upon whether or not highly directional interactions are passivated in the oligomer formation. We expect fitting LLPS measurements to simplified interaction models for mAbs will provide additional insight into the nature of the protein–protein interactions and guide their development for calculating concentrated solution properties.

## Introduction

The number of monoclonal antibodies and antibody-derived products such as antibody–drug conjugates, bispecific antibodies, and antibody fragments under development for a range of medicinal treatments is rapidly growing. For many of these products, the preferred administration route is by subcutaneous injection of a formulation at high drug concentrations often exceeding 150 g/L. At these concentrations, the solutions become more susceptible to undergoing liquid–liquid phase separation (LLPS) into a protein-concentrated phase and a protein-depleted phase. Phase separation introduces additional challenges toward manufacturing antibodies due to causing concentration gradients during formulation, fill and finish operations, storage, and transportation, leading to batch heterogeneity and lowering the aesthetic appeal of the drug product.^1,2^ LLPS has been induced by cooling mAb solutions to refrigeration temperatures^3,4^ or under low ionic strength solutions by changes in pH toward the mAb isoelectric pH (pI)^2,3,5−7^ or addition of multivalent ionic salts or buffers^1,8^ or by adding concentrated salts^9−11^ or polymers, such as polyethylene glycol.^12,13^ The phase boundaries are sensitive to salt type and concentration^3^ as well as the presence of commonly used excipients.^2,7^

There is a need for predicting and controlling the likelihood of phase separation in terms of typical solution conditions used in antibody manufacturing and formulation. LLPS of protein solutions can be rationalized in terms of a colloidal gas–liquid transition, where the same theoretical framework used for predicting gas–liquid transitions of pure fluids can be used for describing the phase transition of protein solutions. The mapping only requires replacing the set of *n*-body interaction potentials with the corresponding set of *n*-body potentials of mean force (pmf).^14^ The effect of the solvent conditions is included implicitly within the pmf, which is a solvent-averaged free energy of interaction. LLPS occurs when the effective protein–protein attractions cross a critical value when altering solvent conditions, such as changing salt concentration or changing temperature. With further increasing the interaction strength, the coexistence curve broadens as the protein concentration difference between the protein-rich and -poor phases increases.

For globular proteins, experimental measurements of phase behavior and protein–protein interactions taken under the same solvent conditions have provided critical insights into the nature and form of the interaction potential. A key parameter determining the location of the binodal is the reduced osmotic second virial coefficient *b*_22_, which relates to an interaction free energy averaged over the relative orientations and separations between a pair of proteins normalized by an excluded volume contribution. Binodals plotted in terms of *b*_22_ collapse on a universal curve for bovine γB-crystallin when precipitated at different ratios of H_2_O to D_2_O ^15^ or for lysozyme when precipitated across a range of solvent and salt conditions at either pH 4.5^16^ or at pH 7.8.^17^ That the phase behavior is insensitive to the details of the interaction potential has been rationalized using simplified spherical models for describing the protein interaction potential. Under typical conditions used for precipitating proteins, the range of the attractive potential compared to the protein diameter is less than 10%.^18−20^ In this limit, the binodal is insensitive to the mathematical form of the interaction potential for isotropic interactions and there is a universal value of the osmotic second virial coefficient at the critical point *b*_22_^c^ ∼ −1.6,^21^ which is near the adhesive hard sphere limit where *b*_22_^c^ ∼ −1.2.^22^ For lysozyme and for γB-crystallin, measured values of *b*_22_^c^ are slightly less than the adhesive hard sphere limit falling in the range of −1.8 < *b*_22_^c^ < −2.9.^16,17,23−26^ The variation in *b*_22_^c^ away from the adhesive limit has been rationalized using anisotropic or patchy models for describing protein–protein interactions.^16,17,24,27,28^ In addition, the binodals predicted using anisotropic potentials are broader and better match experimental phase diagrams than predictions based on isotropic potentials.^28−31^ More recently, the observed variation of binodals when plotted in terms of *b*_22_ for lysozyme has been rationalized using isotropic models.^23^ The study showed that a universal binodal is obtained when the protein size parameter σ is rescaled to account for contributions from repulsive interactions according to the extended law of corresponding states.^32^ However, the values of σ obtained from fitting to the phase behavior could only be rationalized if there exists a long-ranged protein–protein repulsion at moderate salt concentrations where electric double layer forces are sufficiently screened. The authors attributed the extra repulsion to hydration forces, although there is no direct experimental evidence for their existence in protein solutions.

As yet, the utility of using *b*_22_ measurements for predicting the LLPS behavior of mAbs has remained essentially unexplored. We expect mapping phase diagrams in terms of *b*_22_ will not only lead to an improved predictor of phase behavior but provide insight into the nature and form of protein–protein interactions for mAbs. So far, minimal models developed for capturing LLPS of mAbs have been benchmarked against binodals plotted in terms of temperature, which requires assuming a temperature-dependence for the interaction potential.^33,34^ This could lead to misleading findings since protein–protein interactions for mAbs do not exhibit universal behavior. Studies have reported osmotic second virial coefficient *B*_22_ values for a mAb, which are independent of temperature and salt concentration, while for other mAbs, *B*_22_ decreases with decreasing temperature only under conditions where the net protein–protein interactions are attractive.^35,36^ On the other hand, there only exist a small number of studies where protein–protein interactions have been quantified at the same solution conditions that the protein undergoes phase separation. The phase behavior and *B*_22_ values determined by self-interaction chromatography for 8 mAbs under salting-out conditions have been measured as a function of either ammonium sulfate or lithium sulfate concentration.^11,37^ The studies found only a weak correlation between measured values of *B*_22_ and the location of the spinodal line, which delineates the region where phase separation occurs instantaneously. While the critical salt concentrations were not determined, the results suggested significantly stronger net protein–protein attractions are required to induce phase separation than expected for isotropically interacting systems. For the IDEC-152 mAb, the measurements provided an upper estimate for *b*_22_^c^ ≈ −15 assuming the excluded volume contribution to *B*_22_ is around 10–12 mL/g, which is typical for mAbs.^38−41^ On the other hand, for the same IDEC-152 mAb, an earlier self-interaction chromatography study found the value of *b*_22_^c^ is greater than −1 when precipitating with ammonium sulfate.^9^ It is not clear why there is such a large discrepancy in the location of the critical point since there are only slight differences in pH and temperature between the studies. An extensive data set covering the phase boundaries of several mAbs complemented with *B*_22_ measurements by static light scattering has been reported for salting-out conditions.^10^ In that study, the phase boundaries were not reported close enough to the critical point for providing an accurate estimate of *b*_22_^c^ for any of the mAbs.

There is evidence that some mAb solution properties are controlled by anisotropic protein–protein interactions. Modeling of thermodynamic properties has required invoking existence of an oligomeric equilibrium commonly referred to as reversible self-association, which is only possible if protein–protein interactions are highly directional.^42−45^ The presence of oligomers has been detected from neutron spin echo measurements^46,47^ or from analytical ultracentrifugation experiments complemented with dynamic and static light scattering.^48−50^ In addition, in many instances, coarse-grained Y-shaped bead models of antibodies with bead-specific interactions provide better fits to structure factor profiles and osmotic compressibility data than isotropic spherical models or Y-shaped bead models with uniform bead–bead attractions.^41,51−54^ The anisotropic models form transient protein clusters at higher protein concentrations, whose properties have been correlated with the concentrated solution viscosity.^42,43,51,53−57^ The molecular basis for anisotropic interactions has been elucidated particularly in solutions at low ionic strength and pH near the isoelectric point. Under these conditions, many mAbs exhibit anisotropic electrostatic attractions,^38,40,41,53,58^ which have been characterized through rational mutagenesis to disrupt charged patches on mAbs causing significant changes to measured values of either *b*_22_ or the diffusion interaction parameter *k*_D_.^59−64^ This would only be possible if *b*_22_ or *k*_D_ is determined by a small number of directional protein–protein interactions.

As yet, there have been no investigations into the utility of using *b*_22_ as a predictor of LLPS. Predictions based on models covering spheres and particles with convex shapes for varying aspect ratios interacting through weakly anisotropic potentials indicate that *b*_22_^c^ values fall in a relatively small window.^65,66^ For spherical models interacting through sticky patches, the values of *b*_22_^c^ become greater than approximately −3 for models with 5 or greater patches or for 4 patch models, when the fraction of patchy surface coverage exceeds 0.5.^27,30^ While capturing antibody solution properties often requires models reflecting highly directional protein–protein interactions, there are also cases where spherically symmetric models or coarse-grained bead models with uniform bead–bead interactions accurately reproduce thermodynamic properties.^41,43,67^ Furthermore, under conditions dominated by electrostatic attraction, there can still be large large variability in the anisotropic nature of the protein–protein interactions.^42,60,61^ It is then not clear to what extent mAb solutions will exhibit universal values of *b*_22_^c^ and how the values will compare against the critical point solution properties of globular proteins such as lysozyme or γB-crystallin. As such, we have investigated the liquid–liquid phase boundaries in terms of *b*_22_ for three mAbs. For each mAb, phase separation has been induced in low ionic strength solutions at pH 8 and at high ammonium sulfate concentrations. The two different solution conditions are expected to cause different levels of anisotropy in the protein–protein interactions. Low ionic strength conditions are expected to cause anisotropic electrostatic attractions between mAbs, which will be screened at the high ammonium sulfate concentrations required to salt-out the mAbs. In the Supporting Information, we report measurements indicating the temperature dependence of protein–protein interactions depends on the mAb as well as the precipitant type, which further supports the necessity to measure LLPS curves in terms of *b*_22_ rather than temperature.

## Experimental Section

### Relationship between SLS and Protein–Protein Interactions

In a static light scattering experiment, the excess Rayleigh ratio *R*_θ_ is measured as a function of protein concentration *c*_p_. *R*_θ_ is related to the osmotic compressibility of the protein solution (∂Π/∂*c*_p_) according to
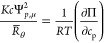
1where *K* is the optical constant, which only depends upon the system configuration and the solvent refractive index, *R* is the gas constant, *T* is temperature, and Ψ_*p*,μ_ = (∂*n*/∂*c*_p_) is the protein refractive index increment. All partial derivatives are taken at constant *T* and the set of solvent and cosolvent chemical potentials. The relationship of static light scattering to protein–protein interactions is often accomplished using the osmotic virial expansion truncated at the second-order term
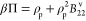
2where *B*_22_^v^ is the osmotic second virial coefficient with units of volume, reduced temperature is β = 1/(*k*_B_*T*) (*k*_B_ is Boltzmann’s constant), and ρ_p_ is the protein number density. An operational form of the light scattering equation is obtained using the osmotic second virial coefficient defined in terms of inverse mass concentration *B*_22_ = *N*_A_*B*_22_^v^/*M*_p_, where *N*_A_ and *M*_p_ correspond to Avogadro’s number and the protein molecular weight, respectively. Using eq 2 for calculating (∂Π/∂*c*_p_) gives
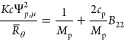
3This approach is only strictly valid when *B*_22_ is determined from the slope of a plot of *KcΨ*_*p*,μ_^2^/*R*_θ_ in the limit of *c*_p_ → 0. From the practical perspective, reducing the uncertainty in the estimate of the experimental slope used to determine *B*_22_ requires carrying out measurements at protein concentrations on the order of g/L. At these protein concentrations and under conditions where protein–protein interactions are sufficiently attractive, thermodynamic properties can become dependent on higher-order interactions. A necessary condition for neglecting higher-order attractive protein–protein interactions is given by *c*_p_*B*_22_ ≫ −1.^68^ Here, we follow the notation that *B*_22_ represents the experimentally derived slope of an osmotic compressibility plot versus protein concentration.

Many mAbs exhibit highly directional protein–protein interactions leading to reversible self-association, which can be captured by representing mAbs as bead models with sticky patches or anisotropic charge distributions^41,51,53^ or through applying chemical association models, where the energetics of patchy interactions are contained within the oligomerization constants.^42,43,45,69^ How patchy interactions relate to the osmotic pressure can be illustrated from the perspective of a reversible chemical association model. For a system undergoing a reversible oligomerization, the virial expansion is given by
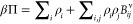
4where the sum is over all oligomer types of size *i* or *j* and the *B*_*ij*_ corresponds to the interaction between an oligomer of size *i* and of size *j*. The main effect of oligomerization at low protein concentrations is to reduce the number of particles, which, in turn, reduces the osmotic pressure. In the Supporting Information, we show the truncated form of eq 4 to second order in the total concentration of associating protein molecules ρ (=∑_*i*_*iρ*_*i*_) is given by^70^
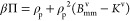
5where *K*^v^ is the dimerization constant in units of volume and *B*_mm_^v^ denotes the *nonspecific* interaction between a pair of monomers, which accounts for all contributions except for the patchy interactions that are buried upon dimerization. In this case, the osmotic second virial coefficient measured from the limiting slope of the osmotic compressibility as *c*_p_ → 0 is given by *B*_22_ = *B*_mm_ – K, where *K* = *N*_A_*K*^v^/*M*_p_ has units of inverse mass concentration. The limiting slope only depends upon contributions from two-body interactions, which is why it does not depend upon any higher order oligomerization constants or any interaction parameters other than *B*_mm_. The truncated form for the multicomponent virial expansion will be accurate only when the mole fraction of dimer is much less than 1, which corresponds to the condition where *c*_p_*K* ≪ 1 which is equivalent to the previous condition that *c*_p_*B*_22_ ≫ −1 under the assumption that nonspecific protein–protein interactions contribute much less to *B*_22_ than the dimerization.

### Relationship of DLS to Protein–Protein Interactions

Dynamic light scattering experiments yield the gradient diffusion coefficient *D*, which controls the decay rate of macroscopic concentration gradients. Because diffusion is driven by chemical potential gradients, the gradient diffusion coefficient is determined by the combination of a thermodynamic and a hydrodynamic term, which is often represented mathematically by *D*/*D*_m,0_ = *Hβ*(∂Π/∂ρ_p_) where *D*_m,0_ is the infinite-dilution value of the monomer diffusion coefficient. The effects of hydrodynamic forces are accounted for in terms of the hydrodynamic function *H*, which is equal to the sedimentation velocity of the protein in an external field normalized by the value at infinite dilution. Expanding the functions to first order in protein concentration gives
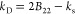
6where *k*_s_ accounts for the effects of two-body interactions on the sedimentation coefficient. The correlation between *b*_22_ and *k*_s_ exists because both parameters relate to an integrated form of the two-body potential of mean force.^71−73^ The two-body hydrodynamic problem has been solved analytically only for the spherical case, which can be used as a good starting point to compare against the experimental data. For the case where all interactions are short-ranged and attractive, the value of *k*_s_ is given by^72,74,75^
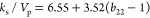
7where *V*_p_ is the volume of a sphere representing the protein in units of mL/g. The first term on the right side of eq 7 (6.55) corresponds to the hard sphere term, which is dominated by the back-flow effect, while the second term accounts for the contribution of short-ranged attractions to *k*_s_. The corresponding equation for *k*_D_ is obtained after substituting the relation for *k*_s_ into eq 6,
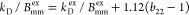
8which is derived assuming that the protein is represented by a sphere with the same excluded volume so that *V*_p_ = *B*_mm_^ex^/4. The contribution of excluded volume interactions is given by *k*_D_^ex^/*B*_mm_^ex^ = 0.36.

It is also instructive to interpret *k*_D_ measurements in terms of a chemical association model. In the Supporting Information, we show that the limiting form for the slope of the diffusion coefficient at low protein concentration, which we denote as *k*_D_, is given by
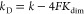
9Here, *k*_D,mm_ is the interaction parameter between a pair of monomers reflecting only the *nonspecific* protein–protein interactions, and *F* is a hydrodynamic factor given by *F* = 1 – *R*_H,m_/*R*_H,d_ where *R*_H,m_ and *R*_H,d_ are the hydrodynamic radii of the monomer and dimer species, respectively. Equation 9 was also derived by Parupudi et al.,^50^ except in their derivation, *k*_D,mm_ is replaced by an average *k*_D_ including monomer–dimer and dimer–dimer interactions. These terms do not appear in our expression because they relate to three-body and four-body interactions. An analogous expression to eq 8 can be derived from noting *B*_22_ = *B*_mm_ – K and assuming that the nonspecific interactions only include contributions from the excluded volume, while all attractions contribute to the dimerization. The resulting expression is given by
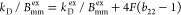
10When representing proteins as spheres, it can be seen that the same correlation occurs irrespective of whether the protein–protein attraction is treated as a physical interaction as represented by eq 8 or in terms of a chemical association model. The ratio of *R*_H,d_/*R*_H,m_ represents the ratio of the friction factor for the dimer versus the monomer. This ratio is equal to 1.392 when the dimer is composed of two tangent spheres,^76^ which leads to 4*F* = 1.13 which should be compared against the factor of 1.12 in eq 8. When rationalizing antibody behavior only in terms of a chemical association model, the value of *B*_22_ – *B*_mm_^ex^ is equivalent to a dimerization constant, which relates to the fraction of proteins that are associated irrespective of whether the association occurs through one directional interaction or is an average over many configurations in which short-ranged attractions are being sampled. The chemical and physical association models are equivalent to each other for spherical models because the friction factor of 2 associating spheres is independent of their relative orientations. The advantage of rationalizing mAb behavior using a chemical association model is that the approach is not restricted to describing antibodies as spheres. The effect of shape appears in the friction factor term *F*, which reflects the hydrodynamic properties of the associated state relative to the monomer. The correlation between *B*_22_ and *k*_D_ will break down when comparing across systems where reversible dimers exhibit different hydrodynamic properties.

### Materials and Methods

Analytical grade sodium chloride was purchased from Thermo Fisher Scientific. Analytical grade Tris and HCl were sourced from Sigma-Aldrich (Dorset, U.K.) and ammonium sulfate from VWR Chemicals (Lutterworth, U.K.). COE-01, COE-07, and COE-19 are IgG1 monoclonal antibodies from AstraZeneca (Cambridge, U.K.). The physical properties of the mAbs are listed in Table 1. Protein–protein interaction measurements and osmotic compressibility data have been reported for COE-19 previously^58,67^ (COE-19 was labeled as mAbB in Singh et al.^58^).

**Table 1:** Biophysical Properties of mAbs^a^

label	mAb type	*M*_w_ (kDa)	ϵ_280_ (mL mg^–1^ cm^–1^)	pI
COE-01	IgG_1_(λ)	144.8	1.56	8.0
COE-07	bispecific IgG_1_	199.9	1.48	8.4
COE-19	IgG_1_(λ)YTE	147.6	1.70	7.7

a *M*_w_ corresponds to the sequence molecular weight, and ϵ_280_ is the extinction coefficent at 280 nm.

Antibody solutions were buffer exchanged by dialysis from their formulation into a 25 mM Tris buffer at pH 8 containing either 200 mM ammonium sulfate or sodium chloride at concentrations of 40 mM for COE-01, 125 mM for COE-07, and 80 mM for COE-19. Sodium chloride concentrations were chosen to prevent phase separation from occurring in solutions at moderate to high mAb concentrations. The protein formulation was placed in 20 kDa MWCO Slide-A-Lyzer dialysis cassettes (Thermo Fisher-Scientific, Loughborough, U.K.). Two 4 h dialysis steps were carried out each using an exchange factor of 1000. After dialysis, samples were filtered through syring-driven 0.22 m filter (Merck Millipore, County Cork, Ireland). The pH was checked and readjusted to pH 8.0 with concentrated acid or base if required. Protein concentrations were measured using a NanoDrop^*C*^ One spectrophotometer (Thermo Fisher-Scientific). For experiments requiring samples at higher protein concentration, the mAb samples were concentrated using vivapsins (Sartorius-Stedim, Goettingen, Germany) in a Sigma 3-16KL swing bucket bench centrifuge (Osterode am Harz, Germany) followed by an additional dialysis step for 4 h using an exchange factor of 1000.

### Simultaneous Static and Dynamic Light Scattering Experiments

SLS and DLS experiments were carried out using a Wyatt MiniDAWN TREOS connected to the CALYPSO II syringe-delivery system from Wyatt using samples with protein concentration varying between 0.4 and 4 g/L following a previously published procedure.^38,58^ For the static light scattering analysis, raw voltages were exported and analyzed in Excel using a value of (∂*n*/∂*c*_p_) equal to 0.185 mL/g. An apparent value for the osmotic second virial coefficient *B*_22_ was obtained from the slope of the plot for *Kc*_p_Ψ_*p*,μ_^2^/*R*_θ_ versus *c*_p_. All error bars reported for the osmotic second virial coefficients were calculated from the standard error in the slope estimation. The intercept of the light scattering plot was used to determine the molecular weights, which are equal to 155.5 ± 2.7 kDa for COE-01, 228.2 ± 2.1 kDa for COE-07, and 149.2 ± 1.1 kDa for COE-19. The values agree well with the expected monomer molecular weight for COE-01 and COE-19. Slightly greater molecular weight values for COE-07 occur irrespective of the salt conditions indicating the samples contain a small amount of irreversibly formed oligomers.

For determination of the mutual diffusion coefficient from the DLS analysis, 10 s acquisitions were used to determine the intensity autocorrelation function, which was fit to a cumulant analysis by the ASTRA software. The minimum delay time used in the fitting was set equal to 0.1 μs, while the maximum delay time was chosen such that the correlation function had decayed to approximately 2% of its initial value. The diffusion coefficients were obtained from averaging the results over the data collected during the delay time. An apparent value for *k*_D_ was obtained from the slope of a plot of *D* versus *c*_p_ according to *D*/*D*_m,0_ = 1 + *k*_D_*c*_p_. Error bars reported on *k*_D_ values were calculated using the standard error in the estimate of the slope. The intercept of the plot *D*_m,0_ was used to determine the hydrodynamic radius of the monomer *R*_H,m_ according to the Stokes–Einstein relationship. The values for *R*_H,m_ averaged over all salt conditions were equal to 5.33 ± 0.03 nm for COE-01, 6.50 ± 0.05 nm for COE-07, and 5.28 ± 0.02 nm for COE-19. The values of *R*_H,m_ were used to calculate the excluded volume contribution to the virial coefficient according to *B*_22_^v^ = (16/3)π*R*_H,m_^3^.^39^

### Coexistence Curve

Coexistence curves were determined using the quench method. 100 μL aliquots of the mAb solution at a concentration of ≈150 mg/mL were placed in Eppendorfs. The concentrated mAb solution was mixed with an appropriate amount of buffer solution (25 mM Tris) and a stock salt solution of sodium chloride or ammonium sulfate with 25 mM Tris, pH 8, to obtain a target salt concentration and a mAb concentration of 90 g/L. The solution was gently mixed by inverting the Eppendorf for a period of 5 min. Samples were then centrifuged in a Heraeus Pico 17 microcentrifuge (Thermo Fisher Scientific) for 5 min at 10 000 rpm. After centrifugation, the protein concentration of the two liquid phases were measured in a NanoDrop^*C*^ One spectrophotometer. When the protein-rich phase was either a gel or precipitate, the dense phase concentration was calculated by carrying out a material balance, which required knowing the volumes of each phase. The protein-poor phase was gently removed and placed in a second Eppendorf tube. The phase volumes were estimated by comparing against Eppendorf standards containing known volumes of liquid.

## Results and Discussion

### Measurement of Protein–Protein Interactions in Terms of *b*_22_

**Figure 1:**
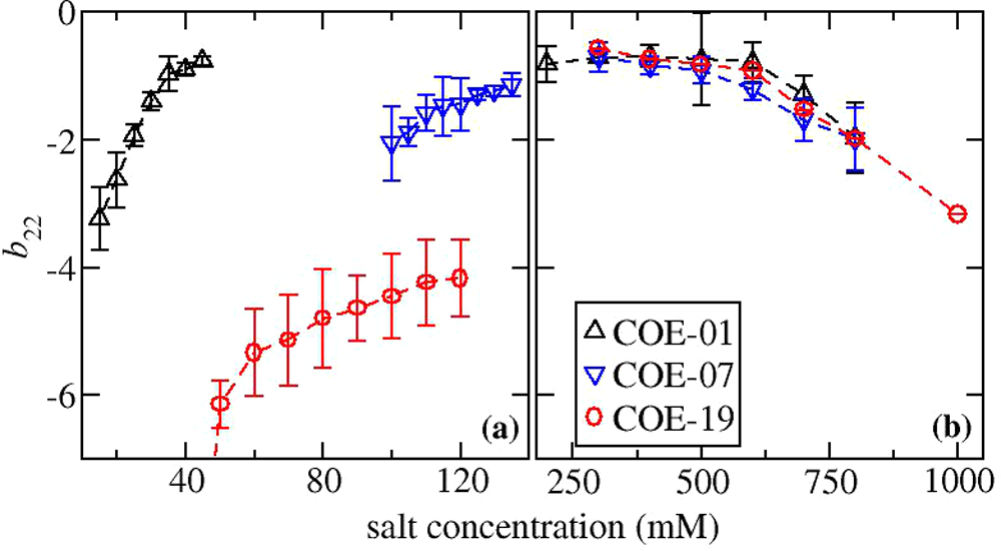
Apparent values for *b*_22_ plotted versus salt concentration for mAbs in solutions with (a) sodium chloride and (b) ammonium sulfate. For COE-19 in solutions with 40 mM NaCl, *b*_22_ = −12.6.

The reduced virial coefficient *b*_22_ for the three mAbs in solutions either with sodium chloride or with ammonium sulfate is shown in Figure 1a or Figure 1b, respectively. The protein–protein interactions observed with sodium chloride versus ammonium sulfate have a different molecular basis. The salt-induced increase in *b*_22_ values for sodium chloride solutions occurs at low ionic strength conditions, where electrostatic interactions are significant. The weakening of protein–protein attractions arises, at least in part, due to screening favorable interactions between surfaces with charge complementarity.^38,40,41,58,77^ It is unlikely that electrostatic interactions alone can cause such a large difference in the protein–protein interactions exhibited by COE-01, COE-07, and COE-19.^40^ Rather the more negative *b*_22_ values exhibited by COE-19 likely arise from combining attractive electrostatics with other short-ranged attractive forces that occur in the same interacting configurations.^58^ The enhancement arises because the probability of sampling a configuration, which also determines the contribution to *b*_22_, is related to a Boltzmann factor of the total interaction free energy.^78,79^ The strong short-ranged attraction for COE-19 is evident from the much lower values of *b*_22_ versus the other mAbs at an ionic strength of 100 mM, where there is moderate electrostatic screening.

Our previous work indicated COE-19 forms small oligomers at protein concentrations below 10 g/L in solutions with 250 mM NaCl and pH 8.^67^ At this condition, the apparent *b*_22_ value of approximately −2.5 is sufficiently less than the value of −0.5 measured with 250 mM ammonium sulfate (see Figure 1b), which corresponds to an ionic strength of 750 mM. This change in *b*_22_ can only be rationalized in terms of screening electrostatic attractions since the other effect of increasing ammonium sulfate concentration is to strengthen short-ranged attractions through a salting-out effect. As such, we suspect the highly directional interactions stabilizing the small oligomers formed by COE-19 at 250 mM NaCl occur in part due to electrostatic attractions, which are strengthened with reducing ionic strength below 100 mM. This explanation is supported by molecular simulation studies examining the ionic strength dependence of *b*_22_ for another mAb,^60^ which indicated the configuration space sampled by the mAb pair at an ionic strength of 315 mM includes attractive electrostatic configurations that become more favored upon decreasing ionic strength.

The *b*_22_ profiles for COE-01, COE-07, and COE-19 in ammonium sulfate solutions overlay each other over the range of salt concentrations between 300 and 800 mM. At first glance, this finding might appear anomalous since there is a large variation in protein–protein interaction measurements reported in the literature for mAbs. However, similar *b*_22_ patterns have been observed for 5 out of 8 mAbs investigated by Lewus et al.,^11^ where the drop in *b*_22_ occurs at an ammonium sulfate concentration around 700 mM. The findings provide insights into the anisotropic nature of the protein–protein interactions under salting-out conditions. If protein–protein interactions are dominated by a small set of highly directional interactions, one would expect them to involve the CDR regions of mAbs, which usually contain the hot spots for protein self-association. However, if this was true, the *b*_22_ profiles should vary between mAbs due to the high variability in the stickiness of the CDR regions. On the other hand, if the protein–protein interaction configuration space is sufficiently diffuse and corresponds to averaging over much of the protein surface, the insensitivity to the CDR regions could be rationalized since they contribute far less to the overall mAb surface area than the constant regions. The observation that salting-out constants of proteins correlate with their retention in hydrophobic interaction chromatography (HIC) also suggests that salting-out is controlled by nonspecific interactions between nonpolar groups on proteins.^80−83^ In addition, some of the developability studies for assessing native-state solubility are based on HIC or salting-out assays indicating that the protein–protein interactions enhanced at high salt concentration have the same molecular basis as much weaker protein–protein interactions occurring at moderate salt concentrations.^84,85^ For many mAbs, the nonelectrostatic contribution to protein–protein interactions can be captured using isotropic interaction models or bead models based on uniform attractions,^41,52,53,67^ which is only possible if the interactions are nonspecific.

### Protein–Protein Interactions in Terms of *k*_D_

**Figure 2:**
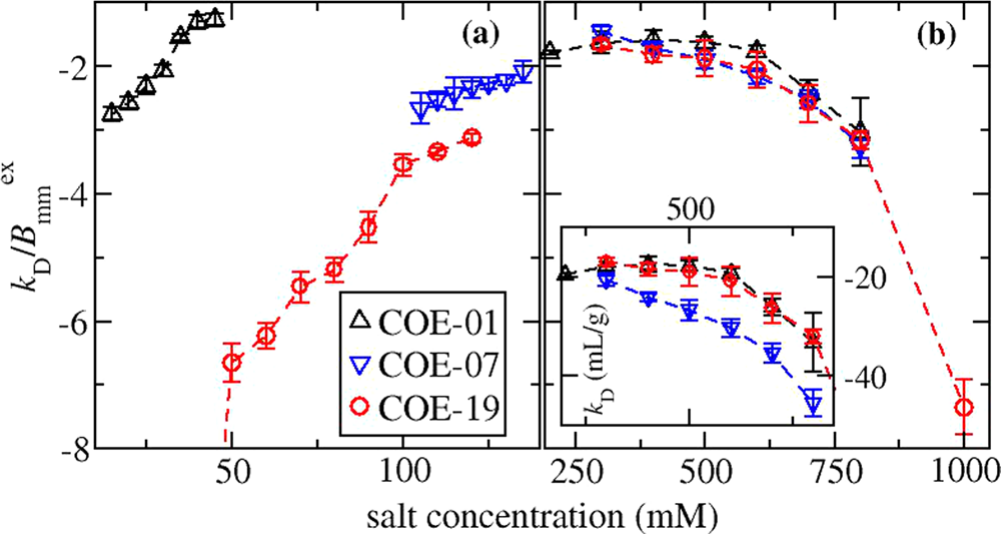
Apparent reduced values for *k*_D_ plotted versus salt concentration for mAbs in solutions with (a) sodium chloride and (b) ammonium sulfate. The inset shows the same data for ammonium sulfate solutions plotted in terms of unreduced values for *k*_D_. For COE-19 in solutions with 40 mM NaCl, *k*_D_/*B*_mm_^ex^ = −12.6.

More insight into the nature of protein–protein interactions can be gained from considering *k*_D_ values, because the parameter contains a contribution from a hydrodynamic term in addition to a thermodynamic term, which directly relates to *b*_22_. In Figure 2, there is a plot containing measured values of *k*_D_ obtained in the same experiment as the corresponding *b*_22_ values at the same solution conditions. The profiles follow the same patterns as the *b*_22_ values with respect to salt concentration. The data are represented in terms of *k*_D_/*B*_mm_^ex^, which is expected to correlate with *b*_22_ according to eq 8. As a comparison, in the inset to Figure 2, there is a plot of the un-normalized *k*_D_ values for the ammonium sulfate solutions, which appear to indicate the protein–protein interactions are more attractive for COE-07 compared to the other 2 mAbs. On the other hand, the profiles when plotted in terms of *k*_D_/*B*_mm_^ex^ indicate the protein–protein interaction profiles are the same for each mAb as also observed in terms of the *b*_22_ measurements. However, there are some discrepancies that are apparent when comparing Figure 2 to Figure 1. The protein–protein interactions for COE-01 and for COE-19 in sodium chloride solutions appear to be more attractive than in ammonium sulfate solutions when characterized in terms of *b*_22_ versus in terms of *k*_D_/*B*_mm_^ex^.

**Figure 3:**
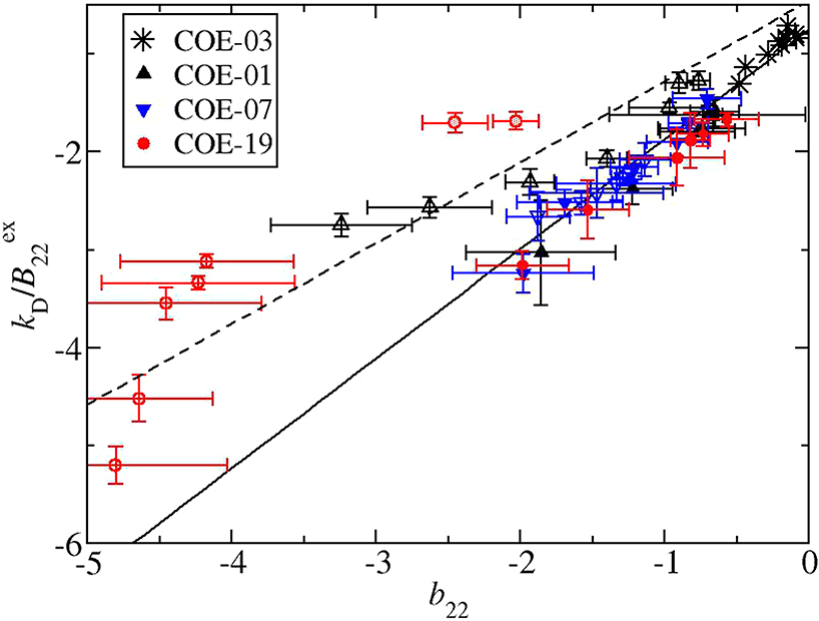
Correlation between measured values of *k*_D_ and *b*_22_ where open or closed symbols correspond to measurements in solutions with sodium chloride or ammonium sulfate, respectively. Shaded red symbols correspond to measurements of COE-19 in solutions with 250 mM NaCl taken from ref (67). Data are also shown for COE-03 which is the mAb studied in ref (38). The full line corresponds to predictions of the Baxter model, and the dashed line corresponds to the formation of dimers that have the same frictional ratio as the monomer.

A plot of the correlation between *b*_22_ and *k*_D_/*B*_mm_^ex^ is shown in Figure 3 along with the theoretical prediction for sticky spheres (eq 8), which is represented as a solid line. The plot also contains data reported for another mAb,^38^ which was referred to as COE-03 in another study,^67^ and the measurements for COE-19 in solutions at either pH 6.5 or pH 8 with 250 mM NaCl.^67^ COE-03 provides an example of a mAb where the thermodynamic properties can be captured using a spherically symmetric interaction potential.^67,74^ The correlation is strongest for the mAbs in ammonium sulfate solutions and for COE-03 where the protein–protein interactions are nonspecific. Indeed the experimentally derived slope close to 1 for the correlation has also been observed with other mAbs under weakly attractive conditions (−2 < *b*_22_ < 1).^35,77,86^

We expect that deviations from the predictions of the sticky sphere model will occur for systems undergoing reversible self-association. According to the sticky sphere model, the average frictional ratio for the ensemble of associated antibody configurations corresponds to a dimer of tangent spheres, where each sphere has the same excluded volume as the mAb.^76^ Notably, the correlation cannot distinguish if the ensemble of associated states corresponds to many configurations or a well defined complex. There are examples where mAbs self-associate into moving units, which are dimers with similar frictional ratios to tangent spheres.^47^ On the other hand, the dimeric sedimentation coefficients for five self-associating mAbs measured by sedimentation velocity experiments exhibit a large variability,^49^ which might also be expected due to diverse hydrodynamic properties exhibited by irreversibly formed dimers.^87,88^ In order to check the sensitivity of the correlation to variation in dimer structural properties, the predictions when mAbs form dimers that have the same frictional ratio as the monomer, which is a commonly used assumption for describing mAb oligomerization,^89^ are included in Figure 3. For ammonium sulfate solutions where protein–protein interactions are nonspecific, there must be significant averaging such that the measured friction factor for the associated states is similar for mAbs. On the other hand, the measurable deviations will arise when mAb interactions are highly directional leading to a small population of associated states with distinct hydrodynamic properties. As an example, clear deviations from the correlation occur for COE-19, which is known to undergo strong reversible self-association in solutions with 250 mM NaCl.^67^ In addition, the results indicate significant reversible self-association for COE-01 with increasing strengths of protein–protein attraction at low sodium chloride concentrations.

The correlation lines shown in Figure 3 are based on the assumption that the measurements of *B*_22_ and *k*_D_ are taken over a concentration range where the solution behavior can be described by two-body interactions. Deviations from the correlation could occur if higher order interactions make significant contributions to the experimentally derived properties. Our previous studies on COE-19 indicated that a reasonable approximation is that *b*_22_ > −2 for neglecting higher-order interactions when measuring the osmotic compressibility in solution up to a protein concentration of 4 g/L.^58^ Because hydrodynamic interactions have a longer range than thermodynamics interactions, it is not clear how this cutoff translates to the interpretation of *k*_D_ measurements. In addition, the analysis is only applicable when there are no longer ranged electrostatic interactions, which is reasonable at pH close to the pI and at high salt concentrations. As such, using deviations from the *b*_22_–*k*_D_ correlation should be used as a guide, rather than an absolute measure, for identifying systems undergoing reversible self-association.

### Phase Diagram Measurements

**Figure 4:**
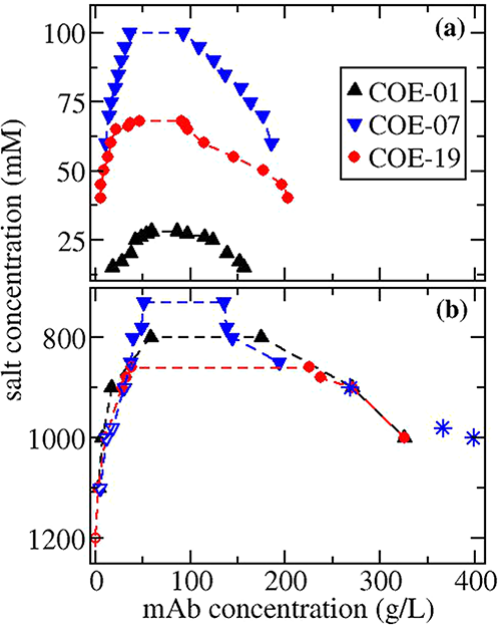
Coexistence curves for mAbs in solutions with (a) sodium chloride or (b) ammonium sulfate. Closed symbols indicate dense phase is a liquid, open symbols indicated dense phase is a gel, hatched symbols indicate dense phase is a solid precipitate. The asterisk symbol indicates the dense phase concentration was determined using a material balance.

Solutions were prepared at a mAb concentration of 100 g/L by mixing a buffered solution at 150 g/L mAb concentration with a stock salt solution. 100 g/L was chosen based on previous reports indicating the concentration is near the critical value. The spinodal and binodal lines join together at the critical point. Within the spinodal, the solution is thermodyamically unstable and phase separation occurs immediately after the sample has been prepared, while the solution is metastable between the binodal and the spinodal. As such, by operating near the critical density, we hoped to avoid any precipitation process which could compete with the LLPS in the metastable region. For all samples exhibiting phase separation, it occurred instantaneously upon mixing the salt solution with the concentrated protein stock solution. LLPS was confirmed by centrifuging the opalescent samples to separate them into two liquid phases. The protein concentration was measured in the dilute phase and in the dense phase to generate a coexistence curve, which is shown in Figure 4. The critical salt concentrations required to induce phase separation are shown in Table 2. At salt concentrations located just outside the phase separation region, the samples remained opalescent, but no phase separation occurred upon centrifugation. With decreasing salt concentration below the critical value, the coexistence curve broadens due to making the protein–protein interactions more attractive.

**Table 2:** Estimated Values for *b*_22_, *k*_D_, and Salt Concentration at the Critical Point for the mAbs in Either Sodium Chloride or Ammonium Sulfate Solutions

	NaCl			(NH_4_)_2_SO_4_		
	COE-01	COE-07	COE-19	COE-01	COE-07	COE-19
*b*_22_^c^	–1.6 ± 0.2	–2.1 ± 0.3	–5.1 ± 0.7	–2.0 ± 0.5	–2.1 ± 0.4	–2.3 ± 0.3
*k*_D_^c^ (mL/g)	–22 ± 1	–41 ± 2	–58 ± 2	–33 ± 5	–38 ± 2	–36 ± 3
*k*_D_^c^/*B*_mm_^ex^	–2 ± 0.1	–2.9 ± 0.2	–5.7 ± 0.2	–3.0 ± 0.5	–2.7 ± 0.2	–3.6 ± 0.3
*C*_salt_^*^ (mM)	28	100	68	800	730	860

A similar approach was used for studying the mAbs in ammonium sulfate solutions. The results are shown in Figure 4b, and critical salt concentrations are provided in Table 2. In contrast to the sodium chloride solutions, the dense phase changes properties with increasing ammonium sulfate concentration. A protein-rich liquid phase was only formed over a small window of ammonium sulfate concentrations near the critical salt concentration. A viscous gel phase was observed when using salt concentrations of 1.1 M and higher for COE-01 and COE-19 and between 0.85 and 1.0 M for COE-07. The gel phase appearance changed from being clear and transparent to slightly cloudy with increasing salt concentration. Visualization under a light microscope indicated the phase is formed by a network of transparent particles, which are likely gel beads due to their nonspherical shapes.^11,37,90^ COE-07 forms an amorphous white precipitate at ammonium sulfate concentrations greater than 1.0 M.

In general, the finding that the mAb concentrations in the dense phase are much higher when ammonium sulfate has been used as the precipitant is consistent with the known LLPS behavior of mAbs. Dense phase concentrations greater than 250 g/L have only been observed for mAbs when LLPS has been induced using ammonium sulfate concentrations greater than 650 mM.^9^ All other LLPS studies have involved using salt concentrations less than 200 mM where the maximum dense phase concentrations are always less than 250 g/L.^1−3,5,91,92^ These differences suggest that the critical point might occur at higher protein densities in concentrated salt solutions. To check if this is true, we measured the opalescence as a function of mAb concentration at NaCl concentrations just greater than the critical values equal to 30 mM, 110 mM, and 70 mM for COE-01, COE-07, and COE-19, respectively, and for each mAb in solutions with 600 mM ammonium sulfate (see Figure S3). For each of the conditions, the maximum opalescence occurs in the protein concentration range of 75–85 g/L, which is between the light and dense phase protein concentrations at the critical salt concentrations. There is no evidence that the critical protein concentrations are greater for the high versus low salt conditions. Previous estimates of the critical concentration from temperature cloud points are 90 ± 9 g/L ^91^ for one mAb, while a value of 100 ± 10 g/L was consistent with the phase diagrams of four mAbs. Similarly, a value of 90 g/L was determined from measuring opalescence as a function of mAb concentration at supercritical temperatures. Critical concentrations much less than 90 g/L are consistent with other LLPS measurements on mAbs where dense phase concentrations near the critical point are as low as 75 g/L.^2,5^

### Location of Critical Point with Respect to *b*_22_

**Figure 5:**
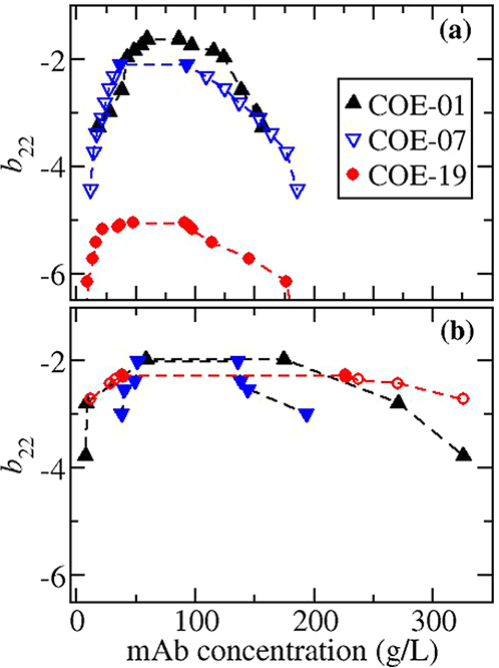
Coexistence curves for mAbs plotted in terms of *b*_22_ in solutions with (a) sodium chloride or (b) ammonium sulfate. Open or closed symbols indicate that values of *b*_22_ have been obtained by extrapolation or interpolation, respectively.

In order to see if *b*_22_ could be used as a predictor for LLPS, the coexistence curves have been replotted in terms of *b*_22_ in Figure 5. The results indicate that the net protein–protein attractions required to cause LLPS are similar irrespective of whether the mAb is precipitated at low or high ionic strength, except for the COE-19 solutions with sodium chloride. While the onset of phase separation occurs at a similar location, the widths of the binodals are much broader for the high versus low salt conditions. Table 2 contains the values for the protein–protein interaction parameters at the critical salt concentrations *b*_22_^c^ and *k*_D_^c^. Except for the solutions of COE-19 at low ionic strength, the values for *b*_22_^c^ fall within a small range between −1.6 and −2.3, which is similar to what has been observed for lysozyme across a range of pH and solvent conditions where – 1.8 < *b*_22_^c^ < – 2.9 ^16,17,23−26^ and for bovine γB-crystallin in solutions where the content of H_2_O to D_2_O varies from to 0 to 100% with *b*_22_^c^ ≈ −2.7.^15^ There is greater variation in the value for *k*_D_^c^/*B*_mm_^ex^, which ranges from −2 to −3.6 (excluding the data for COE-19 at low ionic strength), or for *k*_D_^c^, which falls in the window between −22 and −41 mL/g. Wu et al.^2^ reported similar values for *k*_D_ for another mAb across a few solution conditions where phase separation begins to occur at room temperature. The results indicate that *b*_22_ is a better indicator of phase separation than *k*_D_, which is expected since phase separation and *b*_22_ are related to only the thermodynamic contribution to protein–protein interactions, while *k*_D_ also depends on hydrodynamic interactions.

The irregular shape of an antibody compared to globular proteins leads to a lower density at the critical point,^33^ which opens up the question about how shape influences the value of *b*_22_^c^. This problem has been addressed by computational studies of nonspherical shapes interacting through uniform square-well potentials.^65^ For particles with various convex shapes (cylinders, spherocylinders, ellipsoids), increasing the aspect ratio from 1 (a sphere) to 5 causes a small increase in *b*_22_^c^ from −1.65 to −1.15. Overall, the results suggest that *b*_22_^c^ is relatively insensitive to the protein shape. While antibodies have irregular shapes, their larger aspect ratios should lead to an increase, rather than a decrease, in *b*_22_^c^.

Interaction anisotropy is a key factor in controlling *b*_22_^c^. For patchy sphere models, the value of *b*_22_^c^ is much more sensitive to the number of patches versus the range of the interaction or the size of the patch. With increasing number of patches from 3 to 4 to 5, the values of *b*_22_^c^ increase from approximately −25 to −5 to −3, appearing to approach the isotropic limit.^27^ Interestingly, models based upon nonspecific patch-patch interactions yield the same binodal curves when plotted in terms of *b*_22_ as patch–antipatch representations, which might be more representative of proteins especially under electrostatically controlled conditions. On the other hand, the stability of the binodal depends on the asymmetry of the patch–patch interaction strengths. A small subset of highly favorable interactions will stabilize transient complexes where the low-energy bonds are buried and phase separation can only proceed via the weaker nonspecific interactions.^93,94^ This behavior would be reflected by a lower value of *b*_22_^c^ since the parameter reflects a Boltzmann-weighted average of all protein–protein interactions irrespective of whether or not the interactions contribute to phase separation. Similar patterns of behavior have been observed using simplified mAb models, where the Y-shape has been represented using seven beads with three sticky patches located on the terminal beads corresponding to the tips of the Fab and Fc domains.^34^ The shapes of two experimental binodals were reproduced by the model through varying the patch–patch interaction energetics,^3,91^ yielding approximate values of *b*_22_^c^ equal to −10 and −20 (which is assuming that the model has a similar excluded volume as atomistic representations of mAbs). As with spherical models, increasing the asymmetry in the patch–patch interaction energetics causes a decrease in *b*_22_^c^, which was attributed to the model antibodies forming dimers or chain-like structures that are incapable of phase separation.

The depression of *b*_22_^c^ for COE-19 in sodium chloride solutions provides an example where highly directional attractions are buried by oligomer formation making them unavailable to cause phase separation. For the other mAb/salt conditions, the finding that values of *b*_22_^c^ fall in a small window around −2 suggests that the protein–protein interactions have contributions from at least four or five sticky patches. This is not surprising for solutions with ammonium sulfate where the antibody interactions are expected to be nonspecific, but under low salt conditions, electrostatic attractions are expected to cause directional interactions. In particular large deviations from the *b*_22_–*k*_D_ correlation for COE-01 in sodium chloride solutions suggested reversible self-association. We suspect that in this case the highly directional interactions are not completely buried when forming small oligomers but rather lead to the formation of branched structures, which can grow indefinitely with increasing protein concentration.

### Shape of the Phase Diagram

**Figure 6:**
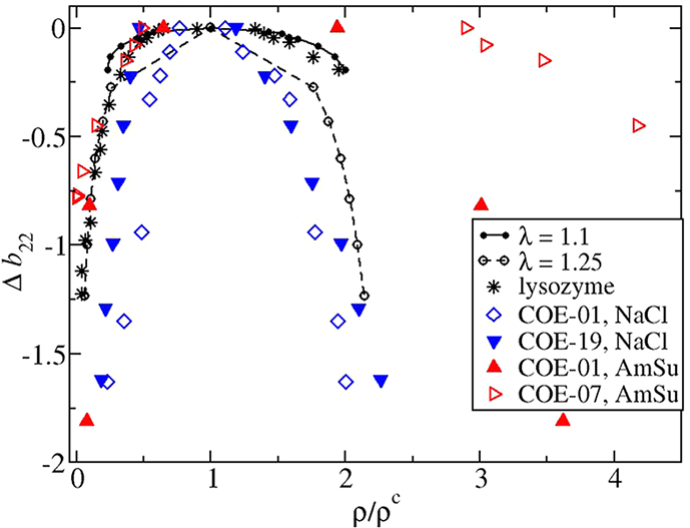
A comparison of experimental coexistence curves plotted in terms of Δ*b*_22_ for the mAbs studied in this work and lysozyme^17^ versus simulated curves for square-well fluids with λ = 1.1 ^95^ or λ = 1.25.^96^ In the legend, ammonium sulfate is abbreviated with AmSu. mAb data sets represented by closed and open symbols indicate the *b*_22_ data were obtained from measured values by extrapolating and interpolating, respectively. .

More insight into the nature of the protein–protein interactions under low ionic strength versus high salt conditions can be gained from considering the width of the binodals. In Figure 6, the binodals for COE-01 under low ionic strength conditions and for COE-07 under salting-out conditions are plotted in terms of Δ*b*_22_ = *b*_22_ – *b*_22_^c^ and compared against experimental data for lysozyme and results obtained from molecular simulations of square-well fluids for two different ranges given by λ = 1.1, 1.25, where λ is the range of the square well normalized by the hard sphere diameter. It has been shown that an extended law of corresponding states is applicable when the binodal is plotted in terns of Δ*b*_22_ for nonspherical convex particles interacting through isotropic and weakly anisotropic interactions.^65,66^ For lysozyme the dilute branch is captured by both models, but the dense branch is better fit to the shorter-ranged potential as the binodal is thinner for λ > 1.25. While the behavior of lysozyme agrees reasonably well with the phase diagrams of model potentials, the binodals of the mAbs deviate greatly from the behavior. Much broader coexistence curves are observed for the mAbs under salting-out conditions, while the binodals are much thinner for the mAbs under low ionic strength conditions.

The finding that the binodal curves are much broader in ammonium sulfate versus sodium chloride solutions does suggest that there are some fundamental differences in the protein–protein interactions under these two conditions. For spherical models, the binodal becomes thinner when making the potential more anisotropic by reducing the number of sticky patches, but this change in potential also leads to a lower *b*_22_^c^ and shifts the critical density to lower values.^27,97,98^ In our study, the lower *b*_22_^c^ only occurs for COE-19 under the low ionic strength conditions, and the critical density does not change much when varying the mAb or the precipitant. Because the difference in phase behavior cannot be explained by spherical models, it is necessary to account for the irregular shape of the antibody. Some critical insights into the nature of the interaction potential have been gained from fitting Y-shaped bead models representing the mAb structure to structure factor profiles. For conditions exhibiting moderate mAb–mAb attraction, structure factor profiles are better captured by anisotropic models where specific attractions are located on the terminal beads representing the tips of the Fab and Fc domains.^51−53^ These models lead to a nearest neighbor peak in the center of mass distribution function at distances greater than the diameter of gyration reflecting the formation of an open network where a pair of antibodies only interact with each other through one or two bead specific attractions.^51^ On the other hand, models with uniform attractions between all beads capture the behavior at weaker levels of protein–protein attractions. At higher protein concentrations, the distribution functions exhibit a peak at a separation of one bead diameter reflecting the formation of densely packed dimers, which maximize all possible interbead attractions. It is conceivable that salting-out interactions are best represented by using Y-shaped models with uniform attractions, which would allow for more tightly packed proteins making the high protein concentrations accessible during LLPS. On the other hand, the thinning of the binodal might arise due to the long-ranged solution structure that is caused by the anisotropic interactions. Along these lines, fitting the behavior to spherical isotropic interaction models requires using longer ranged attractions, on the order of 3–4 nm,^51,53,99^ which is known to cause thinning of the coexistence curve.

## Conclusions

The key finding of our work is that *b*_22_^c^ falls in a small window with *b*_22_^c^ ≈ −2 for five out of the size mAb/salt systems. The result might be expected for ammonium sulfate solutions since protein–protein interactions are expected to be nonspecific and only weakly anisotropic, which is supported by the similar *b*_22_ and *k*_D_ profiles measured for the three mAbs. If interactions are weakly anisotropic, than the same interaction potential will be applicable for describing each of the mAbs, which in turn indicates the binodals will overlay with each other when plotted in terms of the net interaction potential. On the other hand, under electrostatically controlled conditions at low ionic strength, mAbs are known to undergo reversible self-association to form small oligomers. Interestingly, the values of *b*_22_^c^ for COE-07 and COE-01 in low ionic strength solutions are also close to −2, which is especially surprising for COE-01 since deviations from the *b*_22_–*k*_D_ correlation indicate the mAb undergoes reversible self-association. The result that some reversibly associating mAbs follow the universal behavior at the critical point perhaps will provide additional insight into the nature of protein–protein interactions. It might be expected that the critical point is invariant for systems where the highly directional interactions underpinning the reversible self-association allow for plenty of branching points, while deviations occur when the highly directional interactions become passivated, for example, which would happen if the mAbs form dimers or chains, which could not phase-separate unless other protein–protein attractions also occur between the mAbs. As such, we expect these measurements can be used for developing minimal protein interaction models for mAbs to improve upon understanding and prediction of concentrated solution properties and the underlying microstructure.

The practical aspect of this study is that *b*_22_ can be used as an initial guide for assessing whether or not phase separation will occur at higher protein concentrations. More rigorous selection can be achieved by simultaneous measurements of *b*_22_ and *k*_D_ over a range of solution conditions to check if there is any evidence of reversible self-association. Systems following the well-known correlation would be more likely to exhibit a near universal value of *b*_22_^c^. A more promising approach for assessing reversible self-association is through combining analytical ultracentrifugation–sedimentation velocity studies with dynamic and static light scattering, which can be used for separating out the highly directional interactions leading to oligomer formation versus other nonspecific attractions that appear to occur between all mAbs.^49,50^

## Supporting Information Available

Theoretical derivation for the relationship between *k*_D_ and *b*_22_ for protein solutions exhibiting reversible dimerization; opalescence measurements; temperature dependence of protein–protein interactions as characterized by dynamic light scattering (PDF)
