# Supplementary material for: The Relationship between Protein–Protein Interactions and Liquid–Liquid Phase Separation for Monoclonal Antibodies
Source: Mol Pharm. 2023 Apr 11;20(5):2662–74. doi: 10.1021/acs.molpharmaceut.3c00090 (PMC10155204; doi:10.1021/acs.molpharmaceut.3c00090)
Supplement: Supplementary file 1 — mp3c00090_si_001.pdf [file mp3c00090_si_001.pdf]

SUPPLEMETARY INFORMATION: The relationship between protein-protein interactions and liquid-liquid phase separation for monoclonal antibodies: Supplementary Information

Nicole Sibanda, Ramesh Kumar Shanmugam, and Robin Curtis*

E-mail: r.curtis@manchester.ac.uk

Theory

Relationship between B_{22} and k_{D} for reversible self associating proteins

Here, we provide a derivation for the the effect of reversible self association (RSA) on the correlation between the experimentally-derived parameters k_{D} and B_{22}. Within this model, any directional (attractive) interactions contribute to the formation of oligomers, while non-specific attractions and repulsions contribute to the non-ideality terms.

The relationship between RSA and B_{22} can be derived by considering the expansion of the osmotic pressure in terms of protein concentration. Because the second-order term in the osmotic pressure expansion represents contributions only from two-body interactions, we only need to consider self association to form dimers since larger oligomers correspond to higher-order interactions. While the monomer and dimer are treated as separate components, the degrees of freedom is the same as a single component solution, since the concentrations are

related to each other through the dimerization condition. Because measurements are made in terms of the total concentration of all protein molecules, the osmotic pressure should be expressed as a function of the total protein density $\rho = \rho_\mathrm{m} + 2\rho_\mathrm{d}$, where ρ_m and ρ_d are the monomer and dimer number densities (molecules per unit volume).

For the dimerization model, the osmotic pressure is given by

$$\beta\Pi = \rho_\mathrm{m} + \rho_\mathrm{d} + \rho_\mathrm{m}^2 B_\mathrm{mm} + 2\rho_\mathrm{m}\rho_\mathrm{d} B_\mathrm{md} + \rho_\mathrm{d}^2 B_\mathrm{dd} \tag{S1}$$

where the first two terms on the right side of the equation correspond to the ideal solution contribution and the remaining three terms are the virial coefficients including all types of self and cross interactions. The monomer and dimer concentrations need to be determined as a function of ρ using the equilibrium constraint

$$K^\mathrm{v} = \frac{\rho_\mathrm{d}}{\rho_\mathrm{m}^2}, \tag{S2}$$

where K^v is an equilibrium constant with units of volume. Activity coefficient corrections have been neglected in the equilibrium condition, which is a reasonable approximation since an ideal solution is defined in the limit of low protein concentration, which is being considered for the derivation. Substitution of $2\rho_\mathrm{d} = \rho - \rho_\mathrm{m}$ into Equation S2 and solving for ρ_m gives

$$\rho_\mathrm{m} = \frac{1}{4K^\mathrm{v}}\left[(1 + 8\rho K^\mathrm{v})^{1/2} - 1\right], \tag{S3}$$

from which the partial derivative $(\partial\rho_\mathrm{m}/\partial\rho)$ can be derived

$$\frac{\partial\rho_\mathrm{m}}{\partial\rho} = (1 + 8\rho K^\mathrm{v})^{-1/2}. \tag{S4}$$

The taylor series expansion for Equation S3 leads to expressions for the monomer and dimer protein density up to $\mathcal{O}(\rho^2)$, $\rho_\mathrm{m} = \rho - 2\rho^2 K^\mathrm{v}$ and $\rho_\mathrm{d} = \rho^2 K^\mathrm{v}$. Substituting these relations

into Equation S1 gives

$$\beta\Pi = \rho(1-2\rho K^{\mathrm{v}}) + \rho^2 K^{\mathrm{v}} + \rho^2(1-2\rho K^{\mathrm{v}})^2 B_{\mathrm{mm}} + \rho^3 K^{\mathrm{v}}(1-2\rho K^{\mathrm{v}}) B_{\mathrm{md}} + \rho^4 K^{\mathrm{v}2} B_{\mathrm{dd}}. \quad \text{(S5)}$$

All terms $\mathcal{O}(\rho^3)$ represent higher-order interactions than two-body and do not contribute to the limiting slope of the osmotic compressibility plot from which the apparent virial coefficient B_{22} is derived. Keeping terms only up to $\mathcal{O}(\rho^2)$ gives

$$\beta\Pi = \rho + \rho^2(B_{\mathrm{mm}} - K^{\mathrm{v}}), \quad \text{(S6)}$$

which was derived by[1] through statistical mechanical arguments in the framework of McMillan-Mayer solution theory. B_{mm} represents all the intermolecular interactions between a pair of monomers except for the directional interactions involved in dimer formation. For protein exhibiting only short-ranged attraction and no longer-ranged repulsion, this equation provides insight into the meaning of a second virial coefficient. It is arbitrary how a dimer is defined since there will always be multiple configurations that contribute to the population of a dimer. If a dimer is defined as any configuration where the pair of proteins are close enough to sample the short-range attraction, then

$$\beta\Pi = \rho + \rho^2(B^{\mathrm{ex}}_{\mathrm{mm}} - K^{\mathrm{v}}), \quad \text{(S7)}$$

which leads to $B_{22} = B^{\mathrm{ex}}_{\mathrm{mm}} - K^{\mathrm{v}}$. As such, the experimentally derived slope of the osmotic compressibility plot is related to the negative of a dimerization constant.

The parameter k_{D} is obtained from the expansion of the mutual diffusion coefficient D in terms of protein concentration. More generally, D is related to a thermodynamic force and a frictional force through

$$\frac{D}{D_{\mathrm{m},0}} = \beta H \frac{\partial \Pi}{\partial \rho} \quad \text{(S8)}$$

where H is the hydrodynamic function. The hydrodynamic function is related to the effect of

protein concentration on the sedimentation velocity. The sedimentation velocity U is given by a weighted average of the monomer and the dimer sedimentation velocities U_{m} and U_{d} normalized by the infinite dilute value of the monomer sedimentation velocity $U_{\mathrm{m},0}$

$$H = \frac{U}{U_{\mathrm{m},0}} = \frac{\rho_{\mathrm{m}}}{\rho}(1 - k_{\mathrm{s,m}}c_{\mathrm{p}}) + \frac{2\rho_{\mathrm{d}}}{\rho}(1 - k_{\mathrm{s,d}}c_{\mathrm{p}})\frac{U_{\mathrm{d},0}}{U_{\mathrm{m},0}} \tag{S9}$$

where $k_{\mathrm{s,m}}$ and $k_{\mathrm{s,d}}$ are averaged sedimentation interaction parameters, $k_{\mathrm{s,m}} = (\rho_{\mathrm{m}}k_{\mathrm{s,mm}} + 2\rho_{\mathrm{d}}k_{\mathrm{s,md}})/\rho$ and $k_{\mathrm{s,d}} = (\rho_{\mathrm{m}}k_{\mathrm{s,dm}} + 2\rho_{\mathrm{d}}k_{\mathrm{s,dd}})/\rho$ where $k_{\mathrm{s},ij}$ accounts for the effect of component j on the sedimentation of component i.[2,3] Sedimentation velocities at infinite dilution are related according to the Stokes Einstein relation to the ratio of the hydrodynamic radii

$$\frac{U_{\mathrm{d},0}}{U_{\mathrm{m},0}} = 2\frac{R_{\mathrm{H,m}}}{R_{\mathrm{H,d}}} \tag{S10}$$

where the factor of 2 arises because the driving force for sedimentation is proportional to the mass of the particle. The hydrodynamic function can be rewritten using Equation S3 to give

$$H = 4\rho K^{\mathrm{V}}(1 - k_{\mathrm{s,d}}c_{\mathrm{p}})\frac{R_{\mathrm{H,m}}}{R_{\mathrm{H,d}}} + (1 - 2\rho K^{\mathrm{V}})(1 - k_{\mathrm{s,m}}c_{\mathrm{p}}) \tag{S11}$$

and keeping only the terms on the order of ρ gives

$$H = 1 - 2\rho K^{\mathrm{v}} + 4\rho K^{\mathrm{V}}\frac{R_{\mathrm{H,m}}}{R_{\mathrm{H,d}}} - k_{\mathrm{s,mm}}c_{\mathrm{p}}. \tag{S12}$$

All terms $\mathcal{O}(\rho^2)$ and higher represent effects on the hydrodynamic function from higher-order interactions. $k_{\mathrm{s,md}}$ and $k_{\mathrm{s,dd}}$ do not appear in Equation S12 because the parameters reflect three body and four-body interactions. Combining the relations for the hydrodynamic function and the osmotic compressibility ($\beta(\partial\Pi/\partial\rho) = 1 + 2\rho(B_{\mathrm{mm}} - K^{\mathrm{V}})$) gives the final relation for the diffusion coefficient

$$\frac{D}{D_{\mathrm{m},0}} = 1 + (2B_{\mathrm{mm}} - k_{\mathrm{s,mm}})c_{\mathrm{p}} - 4K\left(1 - \frac{R_{\mathrm{H,m}}}{R_{\mathrm{H,d}}}\right)c_{\mathrm{p}} \tag{S13}$$

where the dimerization constant K is defined in terms of inverse mass concentration units ($K = K^{\mathrm{V}} N_{\mathrm{A}}/M_{\mathrm{p}}$). To simplify the equation, we can define a hydrodynamic term $F = 1 - R_{\mathrm{H,m}}/R_{\mathrm{H,d}}$. Using this definition, the measured value of k_{D} becomes

$$k_{\mathrm{D}} = 2B_{\mathrm{mm}} - k_{\mathrm{s,mm}} - 4KF \tag{S14}$$

where the first two terms on the right side are the contributions to k_{D} from non-specific protein-protein interactions and the last term accounts for the effect of reversible self association. Because the measured value of the osmotic second virial coefficient is given by $B_{22} = B_{\mathrm{mm}} - K$, the equation for k_{D} can be rewritten as

$$k_{\mathrm{D}} = k_{\mathrm{D,mm}} + 4F(B_{22} - B_{\mathrm{mm}}) \tag{S15}$$

where $k_{\mathrm{D,mm}}$ is the non-specific contribution to the monomer-monomer interaction parameters. If the dimer is defined as the ensemble of configurations where a pair of proteins are interacting through the short-ranged attraction

$$\frac{k_{\mathrm{D}}}{B_{\mathrm{mm}}^{\mathrm{ex}}} = \frac{k_{\mathrm{D,m}}^{\mathrm{ex}}}{B_{\mathrm{mm}}^{\mathrm{ex}}} + 4F(b_{22} - 1) \tag{S16}$$

In this case, k_{D} is controlled by the hydrodynamic term, which is related to the ratio of dimer to monomer frictional factors (or equivalently hydrodynamic radii). For spheres, the ratio of friction factors is given by $R_{\mathrm{H,d}}/R_{\mathrm{H,m}} = 1.392$, which leads immediately to

$$\frac{k_{\mathrm{D}}}{B_{\mathrm{mm}}^{\mathrm{ex}}} = \frac{k_{\mathrm{D,m}}^{\mathrm{ex}}}{B_{\mathrm{mm}}^{\mathrm{ex}}} + 1.13(b_{22} - 1). \tag{S17}$$

The relationship is almost equivalent to the correlation derived using Batchelor's result assuming monodispersed spheres interacting only through short-ranged attractions.[4]

Methods

Opalescence measurements

Nephelometry experiments were carried out on a NEPHELOstar Plus Microplate Nephelometer (BMG Labtech, Buckinghamshire, UK). Samples of 200 µl were loaded onto 96 clear bottom well plates (Corning, Kaiserslautern, Germany). The NEPHELOstar PLUS uses a laser diode at a wavelength of 635 mm. Experiments were conducted using a beam intensity of 100 % with a 2 mm beam width. Data was collected using BMG Labtech's OMEGA software version 2.41 and analysed using BMG Labtech's MARS data analysis software.

Absorbance experiments were carried out on an Agilent Cary 60 UV-VIS spectrophotometer (Agilent, Stockport, UK). Samples of 500 µl were placed in a micro-cuvette with measurements repeated in triplicate (Appleton Woods Ltd, Birmingham, UK). Data was collected using Agilent Cary WIN UV software (version 5.0.0.999).

Dynamic light scattering experiments as a function of temperature

For temperature ramping experiments, dynamic light scattering was carried out in the DynaPro Plate Reader (Wyatt Technology, Santa Barbara, USA). The Wyatt Plate Reader uses a single 158° detector angle with a laser at a wavelength of 830 nm. 40 µl aliquots of COE-07 were pipetted into 384 well low base, high content imaging plates (Corning, Flintshire, UK). For each measurement, 10 acquisitions of 5 seconds each were taken with measurements done in triplicate. Data was collected and analysed using Wyatt Dynamics software (version 7.8.2.18).

Results

Temperature dependence of protein-protein interactions

A commonly-used approach for causing phase separation is to reduce temperature as many mAbs exhibit an upper consulate solution temperature (UCST). A UCST occurs only if reducing temperature decreases the protein-protein interaction free energy relative to thermal energy. In order to check the likelihood for observing a UCST, we initially measured the temperature dependence of protein-protein interactions in terms of the apparent hydrodynamic radius $R_{\mathrm{H,app}}$, which is related to the mutual diffusion diffusion coefficient through a Stokes-Einstein relationship, for solutions at sodium chloride concentrations just above the critical values and varying protein concentration up to 30 g/L. In Figure S1 there is a plot for the $R_{\mathrm{H,app}}$ values as a function of temperature. There is a pronounced increased in the $R_{\mathrm{H,app}}$ value only for COE-07. For the other two mAbs, the $R_{\mathrm{H,app}}$ values remain constant indicating protein-protein interactions are insensitive to temperature.

For COE-07, the temperature dependence of the k_{D} value for COE-07 in sodium chloride and ammonium sulfate solutions was determined using measurements on samples with protein concentrations up to 10 g/L. The results are shown in Figure S2. As expected, the k_{D} values decrease with reducing temperature in all cases. However, the values for sodium chloride solutions exhibit a much stronger temperature dependence irrespective of the magnitude of the protein-protein attraction. This behaviour should be contrasted with the other two mAbs, which did not exhibit any temperature dependence of protein-protein interactions in either sodium chloride or ammonium sulfate solutions.

Our findings indicate protein-protein interactions become more attractive only for COE-07 upon reducing temperature, while the behaviour for the other two mAbs are insensitive to changes in temperature over the range of 10 to 40°C. Furthermore, for COE-07, the temperature dependence is more pronounced under low ionic strength conditions versus high-salt conditions. Theoretical arguments indicate a stronger temperature dependence occurs when

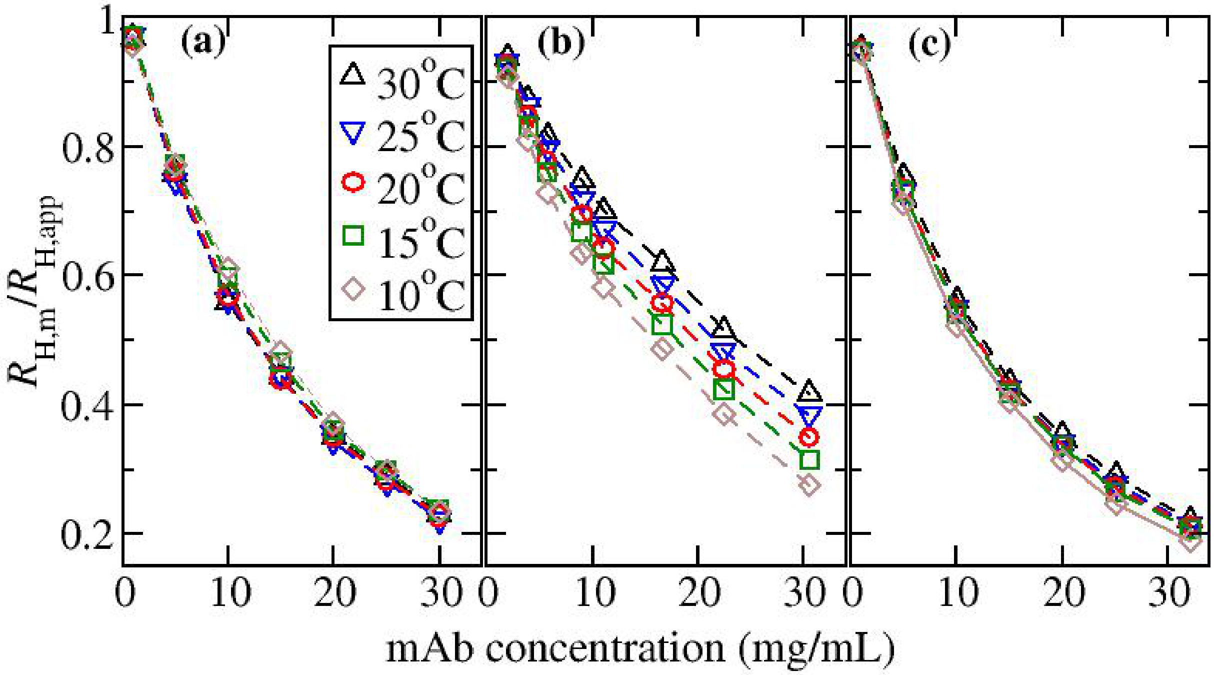

Figure S1: Plots of normalized inverse apparent hydrodynamic radius ($R_{\mathrm{H,m}}/R_{\mathrm{H,app}}$) as a function of protein concentration at different temperatures for (a) COE-01 and 40 mM NaCl, (b) COE-07 and 125 mM NaCl, (c) COE-19 and 85 mM NaCl)

proteins form reversible oligomers,[5] which might provide a partial rationalization for COE-07 behaviour since electrostatic attractions are more anisotropic than salting-out interactions, although this was not apparent from considering the correlation between b_{22} and k_{D}. On the other hand, the strongest reversible self association occurs for COE-17 under low ionic strength conditions, but protein-protein interactions are insensitive to temperature. Woldeyes *et al*[6] has also reported b_{22} as a function of temperature for a pair of mAbs. Analogous to our findings, one of the mAbs does not exhibit a temperature-dependent behaviour, while for the other mAb, lowering temperature only causes a decrease in b_{22} for conditions where the net interaction potential is attractive. Alternatively, LeBrun *et al*[7] found that increasing salt concentration causes the protein-protein interactions to become temperature sensitive, even though under both low and high-salt conditions, the net interaction potential remains repulsive. These results should not be surprising in that under most conditions there will exist an ensemble of interacting configurations that contribute to the net interaction poten-

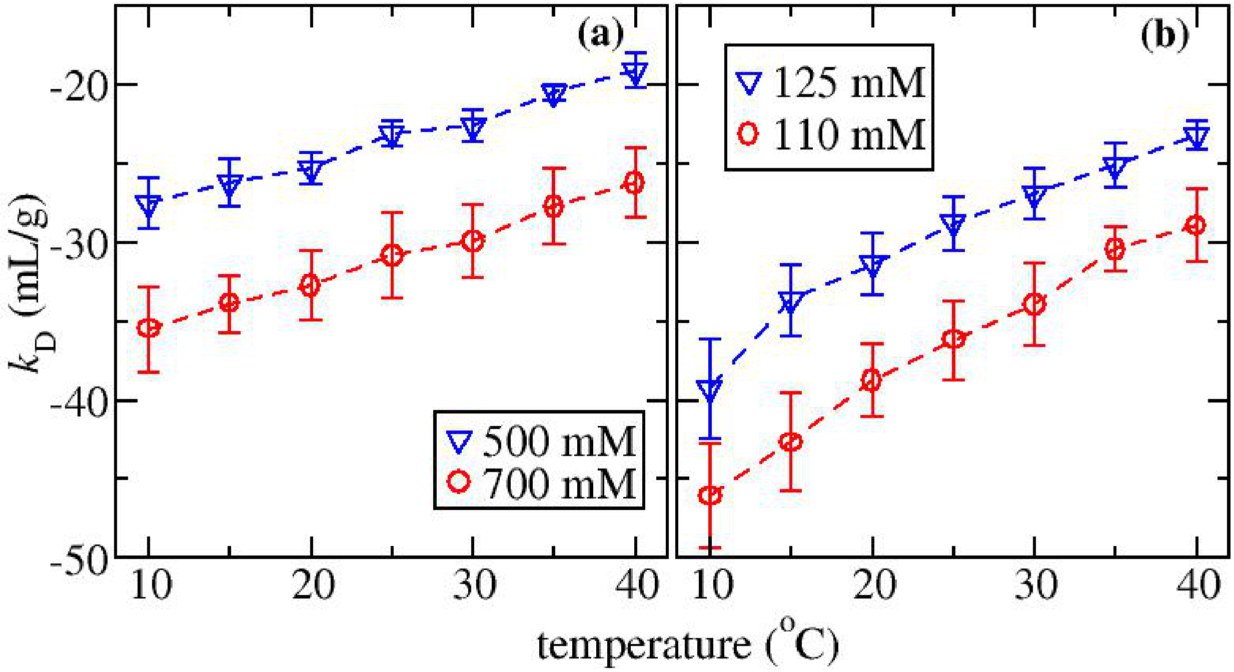

Figure S2: Apparent values for k_D plotted versus temperature for COE-07 in solutions containing either (a) ammonuium sulfate or (b) sodium chloride, where the legend indicates the salt concentration

tial, each which could have a different dependence on temperature. For example, a single point mutation to γD-crystallin causes an inversion of the crystal solubility from normal to retrograde indicating the mutation causes a sticky patchy that becomes more pronounced with increasing temperature,[8] which is opposite to the temperature dependence of the net interaction potential exhibited by the wild type and the mutant. The complicated nature of protein-protein interactions is also evident in other studies. Lysozyme and γ-crystallins, which perhaps are the most well-studied proteins in terms of their phase behaviour, do exhibit reduced solubility with decreasing temperature reflecting enhanced protein-protein attrations,[9–15] while retrograde behaviour has been observed for hemoglobin[16–18], concanavalin A[19] and equine serum albumin[20], and temperature-insensitive interactions exist for proteins such as taumatin[20], α-chymotrypsinogen A[6,21], ribonuclease A[22] and aspartate transcarbamylase[23].

Opalescence measurements

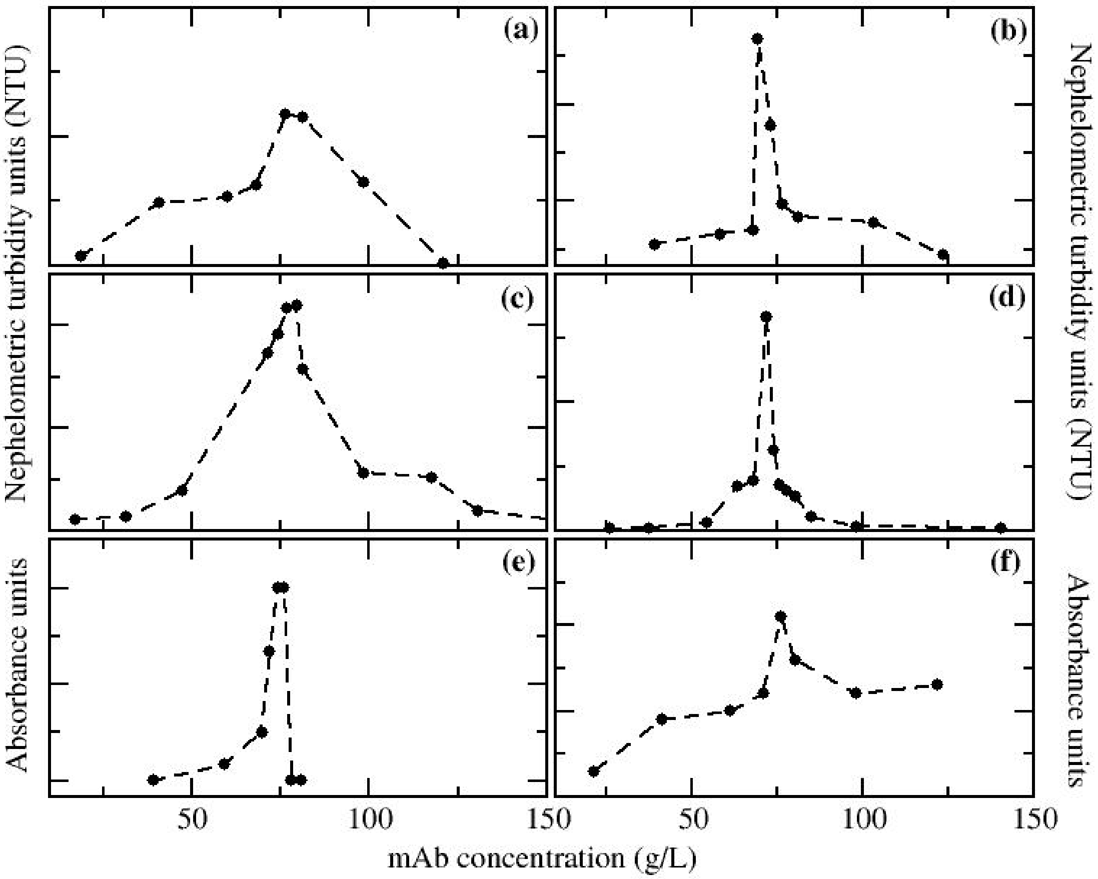

Figure S3: Opalescence was characterized by nephelometry for COE-01 and COE-19 solutions, while absorbance measurements were used for detecting opalescence of COE-07 solutions. (a) COE-01 in solutions with 30 mM NaCl. (b) COE-01 in solutions with 600 mM AmSu. (c) COE-19 in solutions with 70 mM NaCl. (d) COE-19 in solutions with 600 mM AmSu. (e) COE-07 in solutions with 110 mM NaCl. (f) COE-07 in solutions with 600 mM AmSu.

References

(1) Hill, T. L.; Chen, Y. Theory of aggregation in solution. 1. General equations and application to stacking of bases, nucleosides, etc. *Biopolymers* **1973**, *12*, 1285–1312.

(2) Correia, J. J.; Stafford, W. F. Sedimentation velocity: A classical perspective. *Method. Enzymol.* **2015**, *562*, 49–80.

(3) Wright, R. T.; Hayes, D. B.; Stafford, W. F.; Sherwood, P. J.; Correia, J. J. Characterization of therapeutic antibodies in the presence of human serum proteins by AU-FDS analytical ultracentrifugation. *Anal. Biochem.* **2018**, *550*, 72–83.

(4) Batchelor, G. K. Sedimentation in a dilute polydisperse system of interacting spheres. 1. General theory. *J. Fluid Mech.* **1982**, *119*, 379–408.

(5) Roberts, C. J.; Blanco, M. A. Role of anisotropic interactions for proteins and patchy nanoparticles. *J. Phys. Chem. B* **2014**, *118*, 12599–12611.

(6) Woldeyes, M. A.; Qi, W.; Razinkov, V. I.; Furst, E. M.; Roberts, C. J. Temperature dependence of protein solution viscosity and protein-protein interactions: Insights into the origins of high-viscosity protein solution. *Mol. Pharm.* **2020**, *17*, 4473–4482.

(7) Le Brun, V.; Friess, W.; Bassarab, S.; Muhlau, S.; Garidel, P. A critical evaluation of self-interaction chromatography as a predictive tool for the assessment of protein-protein interactions in protein formulation development: A case study of a therapeutic monoclonal antibody. *Eur. J. Pharm. Biopharm.* **2010**, *75*, 16–25.

(8) McManus, J. J.; Lomakin, A.; Ogun, O.; Pande, A.; Basan, M.; Pande, J.; Benedek, G. B. Altered phase diagram due to a single point mutation in human gamma D-crystallin. *Proc. Natl. Acad. Sci. U.S.A.* **2007**, *104*, 16856–16861.

(9) Malfois, M.; Bonnete, F.; Belloni, L.; Tardieu, A. A model of attractive interactions to account for fluid-fluid phase separation of protein solutions. *J. Chem. Phys.* **1996**, *105*, 3290–3300.

(10) Broide, M. L.; Berland, C. R.; Pande, J.; Ogun, O. O.; Benedek, G. B. Binary-liquid

phase-separation of lens protein solutions. *Proc. Natl. Acad. Sci. U.S.A.* **1991**, *88*, 5660–5664.

(11) Gogelein, C.; Nagele, G.; Tuinier, R.; Gibaud, T.; Stradner, A.; Schurtenberger, P. A simple patchy colloid model for the phase behavior of lysozyme dispersions. *J. Chem. Phys.* **2008**, *129*, 085102.

(12) Parmar, A. S.; Muschol, M. Hydration and hydrodynamic interactions of lysozyme: Effects of chaotropic versus kosmotropic ions. *Biophys. J.* **2009**, *97*, 590–598.

(13) Bucciarelli, S.; Mahmoudi, N.; Casal-Dujat, L.; Jehannin, M.; Jud, C.; Stradner, A. Extended law of corresponding states applied to solvent isotope effect on a globular protein. *J. Phys. Chem. Lett.* **2016**, *7*, 1610–1615.

(14) Gibaud, T.; Cardinaux, F.; Bergenholtz, J.; Stradner, A.; Schurtenberger, P. Phase separation and dynamical arrest for particles interacting with mixed potentials-the case of globular proteins revisited. *Soft Matter* **2011**, *7*, 857–860.

(15) Bonnete, F.; Finet, S.; Tardieu, A. Second virial coefficient: Variations with lysozyme crystallization conditions. *J. Cryst. Growth* **1999**, *196*, 403–414.

(16) Vekilov, P. G.; Feeling-Taylor, A. R.; Petsev, D. N.; Galkin, O.; Nagel, R. L.; Hirsch, R. E. Intermolecular interactions, nucleation, and thermodynamics of crystallization of hemoglobin C. *Biophys. J.* **2002**, *83*, 1147–1156.

(17) Serrano, M. D.; Galkin, O.; Yau, S. T.; Thomas, B. R.; Nagel, R. L.; Hirsch, R. E.; Vekilov, P. G. Are protein crystallization mechanisms relevant to understanding and control of polymerization of deoxyhemoglobin S? *J. Cryst. Growth* **2001**, *232*, 368–375.

(18) San Biagio, P. L.; Palma, M. U. Spinodal lines and Flory-Huggins free energies for solutions of human hemoglobins hbs and hba. *Biophys. J.* **1991**, *60*, 508–512.

(19) Wilson, W. W.; DeLucas, L. J. Applications of the second virial coefficient: protein crystallization and solubility. *ACTA Crystallogr. F* **2014**, *70*, 543–554.

(20) Wilson, W. W. Light scattering as a diagnostic for protein crystal growth - A practical approach. *J. Struct. Biol.* **2003**, *142*, 56–65.

(21) Liu, J.; Yin, D. C.; Guo, Y. Z.; Wang, X. K.; Xie, S. X.; Lu, Q. Q.; Liu, Y. M. Selecting temperature for protein crystallization screens using the temperature dependence of the second virial coefficient. *PLoS One* **2011**, *6*, e17950.

(22) Boyer, M.; Roy, M. O.; Jullien, M.; Bonnete, F.; Tardieu, A. Protein interactions in concentrated ribonuclease solutions. *J. Cryst. Growth* **1999**, *196*, 185–192.

(23) Budayova, M.; Bonnete, F.; Tardieu, A.; Vachette, P. Interactions in solution of a large oligomeric protein. *J. Cryst. Growth* **1999**, *196*, 210–219.
